# Global Gene Expression Analysis of Cross-Protected Phenotype of *Pectobacterium atrosepticum*

**DOI:** 10.1371/journal.pone.0169536

**Published:** 2017-01-12

**Authors:** Vladimir Gorshkov, Stanford Kwenda, Olga Petrova, Elena Osipova, Yuri Gogolev, Lucy N. Moleleki

**Affiliations:** 1 Kazan Institute of Biochemistry and Biophysics of Kazan Science Centre of Russian Academy of Sciences, Kazan, Russia; 2 Department of Biochemistry and Biotechnology, Kazan Federal University, Kazan, Russia; 3 Department of Microbiology and Plant Pathology, University of Pretoria, Pretoria, South Africa; 4 Forestry and Agricultural Biotechnology Institute (FABI), University of Pretoria, Pretoria, South Africa; National Renewable Energy Laboratory, UNITED STATES

## Abstract

The ability to adapt to adverse conditions permits many bacterial species to be virtually ubiquitous and survive in a variety of ecological niches. This ability is of particular importance for many plant pathogenic bacteria that should be able to exist, except for their host plants, in different environments e.g. soil, water, insect-vectors etc. Under some of these conditions, bacteria encounter absence of nutrients and persist, acquiring new properties related to resistance to a variety of stress factors (cross-protection). Although many studies describe the phenomenon of cross-protection and several regulatory components that induce the formation of resistant cells were elucidated, the global comparison of the physiology of cross-protected phenotype and growing cells has not been performed. In our study, we took advantage of RNA-Seq technology to gain better insights into the physiology of cross-protected cells on the example of a harmful phytopathogen, *Pectobacterium atrosepticum* (*Pba*) that causes crop losses all over the world. The success of this bacterium in plant colonization is related to both its virulence potential and ability to persist effectively under various stress conditions (including nutrient deprivation) retaining the ability to infect plants afterwards. In our previous studies, we showed *Pba* to be advanced in applying different adaptive strategies that led to manifestation of cell resistance to multiple stress factors. In the present study, we determined the period necessary for the formation of cross-protected *Pba* phenotype under starvation conditions, and compare the transcriptome profiles of non-adapted growing cells and of adapted cells after the cross-protective effect has reached the maximal level. The obtained data were verified using qRT-PCR. Genes that were expressed differentially (DEGs) in two cell types were classified into functional groups and categories using different approaches. As a result, we portrayed physiological features that distinguish cross-protected phenotype from the growing cells.

## Introduction

Adaptation is an important property of living organisms that enables them to survive under stress. In higher organisms, the stress-response is divided into several stages: alarm, resistance and exhaustion. The alarm stage is characterized by the violation of homeostasis (shock) and subsequent induction of specific regulatory systems (e.g. mediated by stress hormones–corticosteroids) provoking mobilization of the endogenous resources to restore homeostasis (contra-shock). During the resistance stage, the cell (organism) restores homeostasis and acquires resistance that lasts until resources are exhausted [1, 2].

Bacteria also have the ability to resist environmental challenges and stress effects despite their relatively simple organization. This ability is related to functioning of specific regulatory networks that change the metabolism of the cells and make them resistant [3]. Well-known components of these networks are bacterial alarmone (p)ppGpp synthesized by RelA or SpoT and stress-induced sigma factor RpoS [4, 5]. These components largely reprogram gene expression that is crucial for survival and formation of so-called cross-protection when one stress factor forms the general resistance to multiple ones. Activation of these stress-related regulatory networks proceeds at the beginning stage of stress response (within a few hours of exposure to stress factors) [6, 7]. Although some of the changes in gene expression that occur during the initial stages of bacterial stress-response were described [8–10], the physiological portrait of the cells that acquired resistance and cross-protective phenotype during the resistance stage of stress-response remains largely unknown.

Cross-protection to multiple stressors in various bacterial species is formed in response to some but not all stress factors [11]. Starvation and acid shock evoke the formation of cross-protection to many if not all different environmental and anthropogenic stressors. Only starvation and acid stresses, but not oxidative or chemical challenge, resulted in increased resistance of *Escherichia coli* and *Salmonella typhimurium* to heat [12, 13]. Starving *E*. *coli* cells were shown to acquire multi-resistance against ionizing radiation, heat and acid shocks, osmotic challenge, and antibiotic treatment [13–17].

Persistence under unfavorable conditions is common for many plant pathogenic bacteria since the life cycle of their host plants usually depends on seasonal changes [18]. Therefore, phytopathogens have adapted to survival in various ecological niches, including water, soil, insects, etc [19–21]. Representatives of harmful soft-rot *Enterobacteriaceace* (*Pectobacterium* and *Dickeya* species) that cause serious plant damages all over the world are known to be able to survive, besides plants, in soil, clouds, sea water, fresh surface water, ground water, insects, and mollusks [22]. Moreover, pectobacteria may apply alternative types of adaptive reactions depending on various exogenous and endogenous factors [23–26]. Some of these reactions were shown to result in the formation of resistance to multiple stress factors: oxidative stress, heat shock, and antibiotic treatment [26]; however, the time necessary for the formation of the cross-protected phenotype was not determined. Altogether, pectobacteria represent suitable model microorganisms for studying microbial adaptation.

The aim of the present study was the elucidation of temporal parameters of cross-protective phenotype formation in *Pba* cultures under stress (starvation) conditions and determination of physiological characteristics that distinguish unstressed growing cells from those that acquired resistance to multiple unfavorable factors as a result of stress-response, in terms of transcriptome profiling using RNA-Seq approach.

## Results

### Cross-protection effect in starving *Pba* cultures

**Fig 1:**
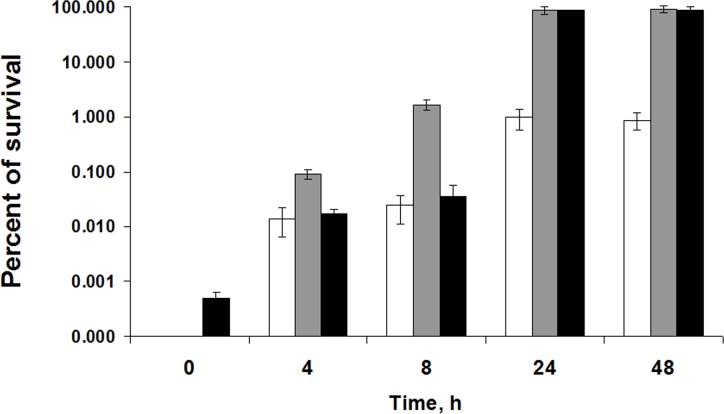
Cross-protective effect in *P*. *atrosepticum* SCRI1043 cells during starvation. *Pba* cells of late log growth phase were transferred to carbon-deficient medium (primary stress). To elucidate the dynamics of the formation of cross-protected phenotype during starvation induced stress, *Pba* cells after 0, 4, 8, 24 and 48 h of starvation were subjected to secondary stresses: 50°C for 5 min (white columns), 2.5 mM H_2_O_2_ for 1 h (gray columns) or 20% NaCl for 1 h (black columns). Cells were plated prior to and right after secondary stresses. The survival of cells starving for 0, 4, 8, 24 and 48 h was assessed by the comparison of cell titer prior and after secondary stress factor exposure. Values are the average ± SD of three biological replicates.

Prior to starvation, *Pba* cells were susceptible to heat shock, oxidative and osmotic stresses: only 0.0005% of the cells were recovered as CFU after exposure to NaCl and no recovery was observed when H_2_O_2_ or heat shock were applied (Fig 1). After four hours of starvation, 0.01, 0.09 and 0.02% of initial cells survived after the exposure to heat shock, H_2_O_2_ and NaCl, respectively, pointing to the formation of cross-protection phenotype in a minor portion of the population. A sharp increase of cross-protective effect was observed after 24 h of starvation: 1.0 ± 0.4%, 85 ± 12.5% and 86 ± 2.7% of cells were recovered by plating after heat shock, oxidative and osmotic stresses, respectively (Fig 1). The cross-protective effect did not increase further after 24 h of starvation. Therefore, the maximal cross-protection effect that is close to 100% (for oxidative and osmotic stresses) is achieved after 24 h of starvation of *Pba* cells.

### RNA-Seq Data Analysis

To characterize whole transcriptome of *Pba* cell after the induction of cross-protective effect, cDNA libraries of growing and 24 h starving cells were prepared. A total of 27.4 and 26.2 million reads were obtained from nutrient rich and starvation conditions, respectively (Table 1). About 98% of total reads were successfully mapped to the reference *Pba* genome sequence using the Bowtie2. In total, approximately 26 million reads were uniquely mapped, with quality scores above Q30.

**Table 1:** Summary of RNA-seq reads mapping to reference genes.

Sample	Total reads	Mapped reads (%)	Uniquely mapped reads (%)
Growth	27439034	27256977 (99.3)	26650668 (97.8)
Starvation	26195940	26004603 (99.3)	25552022 (98.2)

**Fig 2:**
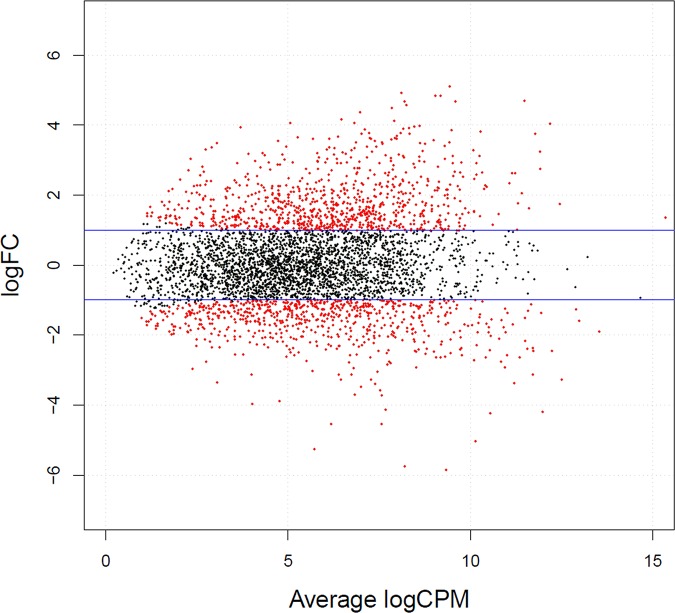
Volcano plot representing *P*. *atrosepticum* SCRI1043 differentially expressed genes under growth-promoting and starvation conditions. Red dots characterize significantly (q < 0.05) DEGs and the blue lines indicate 4-fold changes in the expression level. logFC and logCPM represent the log-**f**old **c**hange in expression and log-**c**ounts **p**er **m**illion for individual genes, respectively.

In total, out of 4626 loci predicted in the genome of *Pba* (NCBI Reference Sequence: NC_004547.2), 4613 transcripts were detected across analyzed samples. The results of the edgeR analysis indicated that 1648 genes had statistically significant differences in expression levels (FDR <0.05) (S1 Table). Among them, 610 were up-regulated and 1038 were down-regulated after the formation of cross-protected phenotype under starvation conditions (Fig 2 and S1 Table). Besides, 265 additional transcripts were revealed using old genome assembly (NC_004547.1); among them additional 29 down-regulated and 55 up-regulated DEGs were found.

### Verification of RNA-seq data

**Fig 3:**
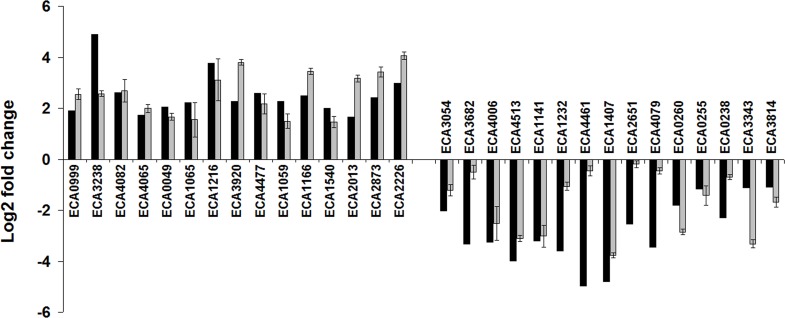
Verification of RNA-Seq data. Expression levels of genes in cross-protected *P*. *atrosepticum* cells determined by RNA-Seq (black columns) and qPCR (grey columns) respective to unstressed growing cells (equal to zero).

To validate RNA-Seq data, the expression levels of 15 up- and 15 down-regulated genes (according to RNA-Seq) under starvation belonging to different pathways were assessed by qPCR analysis. The results showed that qPCR data were in good agreement with those of RNA-Seq (Spearman’s correlation coefficient 0.78, P-value 1.76 × 10^−6^) (Fig 3).

### Classification of DEGs into functional gene modules and pathways

**Fig 4:**
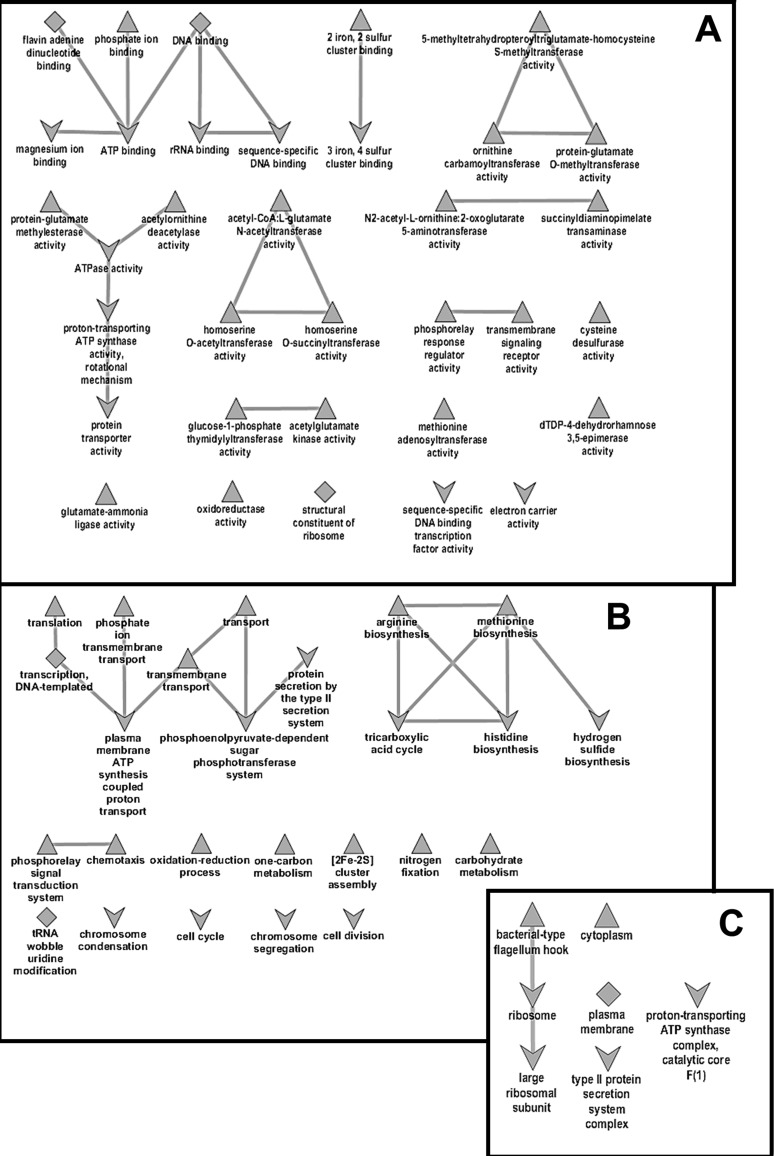
Gene ontology (GO) analysis of differentially expressed genes. Networks composed of statistically significant (FDR < 0.05) non-redundant GO terms associated with up-regulation (triangles), down-regulation (arrowheads), or both up- and down-regulation (diamonds) of expression of genes related to molecular functions (A), biochemical processes (B), and cellular components (C) in adapted *P*. *atrosepticum* cells relative to the growing ones. Figures were visualized by REVIGO software.

To gain better insight into physiological processes that distinguish non-adapted growing *Pba* cells from those that acquired cross-protected phenotype as a result of starvation induced stress response, the classification of genes into functional gene modules and pathways was performed. Gene ontology (GO) analysis of differentially expressed genes (DEGs) revealed 76 gene categories: 51 up- and 35 down-regulated (ten categories were present among both up- and down-regulated) (Fig 4, S2 Table and S3 Table). 330 DEGs were not classified into GO categories.

KEGG mapping identified 681 of DEGs of 1649, including 156 up-regulated and 525 down-regulated genes that were mapped into 21 pathways. More than 58% of revealed DEGs were absent in KEGG pathways. These unmapped DEGs together with those that were mapped into broad categories (“Metabolic pathways”, “Microbial metabolism in diverse environment” and “Biosynthesis of secondary metabolites”) were manually classified into gene modules and pathways using Uniprot, MiST2.2, Pfam, PROSITE, ICEberg, Ecogene, String, [27], TAD, dndDB, SMART, HAMAP, EMBL-EBI, REBASE, InterPro databases (marked in tables with star) (Table 2). Ten new gene modules (One-component systems, Horizontally acquired islands, Response to environmental stress, Protein modification, Cell envelope, Biofilm formation, Conjugation and phage-like proteins, Sigma-anti-sigma factors, Other proteins, Uncharacterized proteins) were distinguished using manual classification and 43 pathways were signified using this approach (*Amino acid metabolism/biosynthesis, *Antioxidant defense, *Biofilm formation, *Carbohydrate metabolism, *Carbon metabolism, *Cell division, *Cell envelope biogenesis, *Chaperone protein, *Chromosome condensation, *Conjugation and phage-like proteins, *Coronofacic acid synthesis, *Cytochrome B- and C-type biogenesis, *DND system, *Glutamate metabolism, *Hydrolases of cell wall, *Membrane proteins, *Mobile genetic elements, *Motility, *Nitrogen metabolism, *Nucleotide metabolism, *One component system, *Other proteins, *Other transporters, *Phage-like proteins, *Polyketide metabolism, *Protein metabolism, *Replication and repair, *Response to environmental stress, *Restriction-modification, *Ribosome, *RNA restriction enzyme, *S-adenosyl-L-methionine biosynthesis, *Sigma-anti-sigma factors, *Stress proteins, *Toxin-antitoxin systems, *Transcriptional regulators, *Transporters, *tRNA, *Two component system, *Uncharacterized proteins and proteins of unknown function, *Virulence, *Lipopolysaccharide biosynthesis) (S4 Table, S5 Table).

**Table 2:** Number of DEGs assigned to a particular pathway by means of GO, KEGG and manual classification using different databases.

Database	Up-regulated DEGs	Down-regulated DEGs	Total number
GO	367	868	1235
KEGG	156	525	681
**Manually**			
Uniprot	218	260	478
Bell et al., 2004	103	103	206
MiST	54	17	71
Pfam	38	33	71
PROSITE	25	10	35
ICEberg	19	12	31
Ecogene	12	6	18
String	18	0	18
TAD	18	0	18
dndDB	6	0	6
SMART	4	2	6
HAMAP	1	4	5
EMBL-EBI	4	0	4
REBASE	4	0	4
InterPro	2	0	2

Taken together, Gene Ontology, KEGG and manual classification using different databases (Table 2) allowed division of the revealed DEGs into 33 gene modules; among these gene modules 136 pathways were distinguished (S4 Table and S5 Table).

### Up-regulated genes in the cross-protected *Pba* phenotype

GO analysis of the 610 up-regulated genes revealed 51 subcategories: 22 biological process subcategories, 3 cellular component subcategories, 26 molecular function subcategories (Fig 4 and S2 Table). The largest subcategories in the biological process category were DNA-templated transcription (GO:0006351), chemotaxis (GO:0006935), DNA-templated regulation of transcription (GO:0006355), tRNA wobble uridine modification (GO:0002098), arginine biosynthetic process (GO:0006526), phosphorelay signal transduction system (GO:0000160) and methionine biosynthetic process (GO:0009086), which constituted from 1.96 to 7.37% of all up-regulated genes. In the cellular component category, the largest subcategories were cytoplasm process (GO:0005737) and plasma membrane (GO:0005886) with 11.97% and 3.77% of all up-regulated genes, respectively. DNA binding (GO:0003677), signal transducer activity (GO:0004871), oxidoreductase activity (GO:0016491), flavin adenine dinucleotide binding (GO:0050660), transmembrane signaling receptor activity (GO:0004888), 5-methyltetrahydropteroyltriglutamate-homocysteine S-methyltransferase activity (GO:0003871) were the most abundant GO terms in the molecular function subcategory (10.82%, 4.75%, 3.11%, 2.12%, 1.48% of all up-regulated genes, respectively).

Up-regulated DEG were grouped into 10 categories (p value <0.05) according to KEGG pathways. Several pathways were significantly induced in cross-protected *Pba* cells: Bacterial chemotaxis (eca02030) (58.1% of genes in KEGG pathway), Arginine biosynthesis (eca00220) (50.0%) and Two-component system (eca02020) (24.7% of genes in KEGG pathway). Up-regulation of 2.2–8% genes in the pathway were observed for Metabolic pathways (eca01100), Microbial metabolism in diverse environments (eca01120), Carbon metabolism (eca01200), Biosynthesis of secondary metabolites (eca01110), ABC transporters (eca02010), Purine metabolism (eca00230) and Pyruvate metabolism (eca00620) (Table 3).

**Table 3:** KEGG pathway analysis of *P*. *atrosepticum* genes up- and down-regulated in cross-protected cells compared to growing ones (p-value ≤0.05).

KEGG path ID	KEGG term	% genes involved in pathway,	p-value
**Up-regulated**			
eca01100	Metabolic pathways	6,40%	1,75E-08
eca02030	Bacterial chemotaxis	58,10%	1,03E-07
eca01120	Microbial metabolism in diverse environments	5,40%	0,00081
eca02020	Two-component system	24,70%	0,00179
eca01200	Carbon metabolism	3,70%	0,00298
eca00220	Arginine biosynthesis	50,00%	0,00521
eca01110	Biosynthesis of secondary metabolites	8,00%	0,00779
eca02010	ABC transporters	7,60%	0,00899
eca00230	Purine metabolism	4,10%	0,03058
eca00620	Pyruvate metabolism	2,20%	0,03758
**Down-regulated**			
eca01100	Metabolic pathways	34,90%	9,35E-09
eca00190	Oxidative phosphorylation	69,80%	1,18E-05
eca01120	Microbial metabolism in diverse environments	40,00%	3,59E-05
eca03010	Ribosome	52,60%	4,26E-05
eca01200	Carbon metabolism	42,20%	0,00112
eca00051	Fructose and mannose metabolism	60,60%	0,00115
eca03070	Bacterial secretion system	45,20%	0,00418
eca00562	Inositol phosphate metabolism	80,00%	0,01124
eca00010	Glycolysis / Gluconeogenesis	45,50%	0,01519
eca00520	Amino sugar and nucleotide sugar metabolism	45,50%	0,01519
eca00020	Citrate cycle (TCA cycle)	51,70%	0,01792
eca01110	Biosynthesis of secondary metabolites	30,70%	0,01878
eca00920	Sulfur metabolism	48,50%	0,02538
eca00680	Methane metabolism	50,00%	0,03649
eca00500	Starch and sucrose metabolism	45,50%	0,03908
eca00630	Glyoxylate and dicarboxylate metabolism	46,40%	0,04366

Up-regulated genes with manually assigned descriptions were involved in such pathways as *Motility, *One-component system, *Two component system, *Glutamate metabolism, *S-adenosyl-L-methionine biosynthesis, *Carbohydrate metabolism, *Replication and repair, *Restriction-modification, *DND system, *Chromosome condensation, *Transcriptional regulators, *Ribosome, *RNA restriction enzyme, *tRNA, *Virulence, *Toxin-antitoxin, *Nucleotide metabolism,*Polyketide metabolism, *Horizontally acquired islands, *Transporters, *Stress proteins, *Antioxidant defense, *Peptidases, *Hydrolases of cell wall, *Membrane proteins, *Conjugation and phage-like proteins, *Type IV secretion system, *Integrated plasmids, *Other proteins, *Uncharacterized proteins and proteins of unknown function and *Other transcripts (Table 4 and S4 Table).

**Table 4:** Modules and pathways of genes expressed differentially in *P*. *atrosepticum* during growth in nutrient rich medium and after the formation of cross-protected phenotype under starvation conditions. Manually assigned modules and pathways are marked with stars.

	Gene module	Starvation repressed			Starvation induced		
		Number of genes	Pathways		Number of genes	Pathways	
1.	Carbohydrate metabolism	95	*Carbohydrate metabolism, eca00010,eca00020, eca00040, eca00051, eca00052, eca00053, eca00500, eca00520, eca00562, eca00620, eca00630, eca00640; eca00650		13	eca00020,eca00660, eca00040, eca00051, eca00520, eca00562, eca00620, eca00630, *Carbohydrate metabolism	
2.	Carbon metabolism	47	eca01200, *Carbon metabolism		4	eca01200	
3.	Amino acid metabolism	38	eca00250, eca00260, eca00300, eca00360, eca00450, eca00473, eca00480, eca01007, eca01230, Cysteine and methionine metabolism eca00270, *Amino acid biosynthesis		37	eca01230, eca00250, eca00290,eca00330, eca00380, eca00400, eca00440, eca01007, *Glutamate metabolism	
						Arginine biosynthesis eca00220	
						Cysteine and methionine metabolism eca00270, *S-adenosyl-L-methionine biosynthesis	
4.	Lipid metabolism	15	eca00061, eca00561, eca00564, eca00591, eca01040, *Lipid metabolism		3	eca00564, eca00561	
5.	Nucleotide metabolism	24	eca00230, eca00240, *Nucleotide metabolism		5	eca00240, eca00230, *Nucleotide metabolism	
6.	Biosynthesis of secondary metabolites	39	eca01110		24	eca01110	
7.	Metabolism of cofactors and vitamins and terpenoids and polyketides	22	eca00523, eca00730, eca00750, eca00760, eca00780,eca00790, eca01053; eca01054		8	eca00790, eca00523, eca00130, eca00785, *Polyketide metabolism	
8.	Nitrogen metabolism	11	eca00910, *Nitrogen metabolism		3	eca00910	
9.	Sulfur metabolism	18	eca00920, eca04122		5	eca04122, eca00920	
10.	Phosphotransferase system	18	eca02060		4	eca02060	
11.	Oxidative phosphorylation	37	eca00190, *Cytochrome B- and C-type biogenesis		0	-	
12.	Transcription	19	eca03000, eca03021		40	eca03000, eca03021	
			*Transcriptional regulators			*Transcriptional regulators	
13.	Translation	86	eca03012, eca00970, eca03009, eca03010, eca03016, eca03019		11	eca03019, eca03009, eca03010, eca03012, eca03016, *RNA restriction enzyme	
14.	*Protein modification	29	eca01001, eca01002, *Protein metabolism		2	*Peptidases	
15.	DNA metabolism and modification	43	Replication and repair: eca03030, eca03400, eca03410, eca03430, eca03440		23	Replication and repair: eca03030, eca03032, eca03400, eca03410, eca03420, *Replication and repair	
			Other: eca03036, *Cell division, *Chromosome condensation			Other: eca03036 *Restriction-modification, *DND system, *Chromosome condensation	
16	*Conjugation and phage-like proteins	22	*Phage-like proteins, *Integrative and conjugative elements (*HAI2), *Type IV secretion system (*HAI7)		79	*Phage-like protein, *Integrative and conjugative elements (*HAI2), *Type IV secretion system (*HAI7), *Integrated plasmids (HAI13)	
17.	Secretion system, *Virulence	76	eca00536, eca01008, eca02044, eca03070, *Coronofacic acid synthesis, *Virulence		12	eca03070, eca02044, *Virulence	
18.	*Response to environmental stress	20	*Stress proteins, *Antioxidant defense		32	eca03110, eca03036, *Stress proteins, *Antioxidant defense	
19.	Prokaryotic defense system	8	eca02048, *Toxin-antitoxin		27	eca02048, *Toxin-antitoxin, *Restriction-modification, *DND system	
20.	Bacterial chemotaxis and motility	17	Bacterial chemotaxis: eca02030, eca02035		44	Bacterial chemotaxis: eca02030, eca02035	
			Flagellar assembly: eca02040			Flagellar assembly: eca02040, *Motility	
21.	*Biofilm formation	4	eca01003, eca01005		19	*Biofilm formation	
22.	*Cell envelope	71	Lipopolysaccharide biosynthesis: eca01005, eca00540, Peptidoglycan biosynthesis: eca00550, eca01003, *Cell envelope biogenesis, *Membrane proteins		48	Lipopolysaccharide biosynthesis: eca01005, *Hydrolases of cell wall, *Cell envelope biogenesis, *Membrane proteins	
23.	Transporters	151	eca02000, eca02010, *Other transporters		48	eca02000, eca02010, *Other transporters	
24.	Chaperones	17	eca03110, *Chaperone protein		5	eca03110	
25.	*One-component system	17	*One-component system		54	*One-component system	
26.	Two-component system	25	eca02020		41	eca02022, eca02020, *Two component system	
27.	*Sigma-anti-sigma factors	5	*Sigma-anti-sigma factors		7	*Sigma-anti-sigma factors	
28.	Metabolic pathways	237	eca01100		44	eca01100	
29.	Microbial metabolism in diverse environments	82	eca01120		11	eca01120	
30.	*Horizontally acquired islands	117	HAI	Number of genes	140	HAI	Number of genes
			HAI1	0		HAI1	3
			HAI2	13		HAI2	24
			HAI3	1		HAI3	3
			HAI4	1		HAI4	7
			HAI5	20		HAI5	2
			HAI6	10		HAI6	8
			HAI7	3		HAI7	32
			HAI8	23		HAI8	13
			HAI10	5		HAI10	2
			HAI11	0		HAI11	2
			HAI12	1		HAI12	5
			HAI13	0		HAI13	20
			HAI14	10		HAI14	7
			HAI15	2		HAI15	2
			HAI16	22		HAI16	10
			HAI17	7		HAI17	0
31.	*Other proteins	44	*Other proteins		43	*Other proteins	
32.	*Uncharacterized proteins	79	*Uncharacterized proteins and proteins of unknown function		75	*Uncharacterized proteins and proteins of unknown function	
33.	*Other transcripts	12	*Other transcripts		23	*Other transcripts	

* Manually assigned modules and pathways.

The summation of the results of automatic analysis using GO and KEGG and manual gene classification using various databases revealed functional gene modules and pathways induced in the cross-protected phenotype (Table 4 and S4 Table). Several defense-related genes, including those for universal stress protein UspB, cell division inhibitor SulA, DNA protection during starvation protein Dps, heat and acid shock proteins DjlA and Ars were up-regulated. Besides, 25 genes participating in antioxidant defense, including putative catalase ECA1216, hydrogenase-4 complex, thioredoxins ECA1267 and *trxC* were induced in starving *Pba* cells.

A group of the genes related to biofilm formation was up-regulated in cross-protected phenotype (Table 4 and S4 Table). This group includes genes whose products are engaged in the synthesis or transport of capsular polysaccharides and several genes encoding regulators involved in biofilm formation: YbaJ and BssS [28, 29], and diguanylate phosphodiesterases (ECA1841, ECA2008, ECA3271, ECA3549) and diguanylate cyclases (ECA3199, ECA3270, ECA3374, ECA3886) engaged in the synthesis of cyclo-di-GMP that was shown to induce biofilm formation in *Pba* [30].

Many conjugation-related genes were up-regulated in *Pba* after the formation of cross-protected phenotype under starvation conditions. Among these genes are those located within HAI-7 that was assumed to encode a full conjugative transfer system [27, 31], HAI-2 and HAI13 that encode integrative and conjugative elements and putative integrated plasmids participated in conjugation, respectively [32] (S4 Table). Several genes belonging to a group of prokaryotic defense system, including restriction-modification, DND and toxin-antitoxin system were up-regulated under starvation (Table 4 and S4 Table).

Activation of several pathways related to amino acid metabolism occurred in adapted cells. Operon *argADECBH* for arginine and citrulline biosynthesis was up-regulated during the starvation period. The expression levels of the genes for methionine synthesis from homocysteine (ECA0181 and ECA3126), methionine synthase (ECA1113) as well as *metF*, which product catalyses the conversion of 5,10-methylenetetrahydrofolate to 5-methyltetrahydrofolate, a cosubstrate for homocysteine remethylation to methionine, were also increased in adapted cells. Almost all genes for methionine salvage cycle were up-regulated under starvation: methylthioribose kinase (ECA3476), methylthioribose-1-phosphate isomerase (ECA3477), acireductone dioxygenase 1 (ECA2986). Transport of methionine was also activated partially in starving bacterial cells: methionine import ATP-binding protein MetN1 (ECA2063), phosphate transport system permease protein (ECA4475), lipoprotein (D-methionine transport system substrate-binding protein, ECA1498) (Table 4 and S4 Table).

Adapted cells had an increased level of transcripts related to chemotaxis. Twenty one of 36 genes for methyl-accepting chemotaxis proteins along with coupling protein CheW, two component kinase CheA, methyltransferase CheR, methylesterase CheB and response regulator CheY, which phosphorylated form is able to interact with flagellar motor were up-regulated under starvation. Genes for stator component of motor (*motA* and *motB*) and flagellar hook-associated proteins (*flgK*, *flgL*, *fliR* and *fliD*) were also up-regulated. Herewith, genes encoding flagellar assembly proteins and genes assigned to bacterial motility (pathways eca02035 and eca02040) were down-regulated. Additionally, gene encoding negative regulator of flagellin synthesis FlgM (ECA1700) was up-regulated (Table 4 and S4 Table).

Many genes related to signaling and signal transduction were differentially expressed in growing cells cross-protected *Pba* phenotype. Out of 292 genes encoding one-component regulatory systems (according to database MiST 2.2.) 56 and 17 were up- and down-regulated under starvation, respectively. The level of transcripts for genes encoding proteins for biosynthesis of secondary messenger cyclo-di-GMF (diguanylate cyclases and diguanylate phosphodiesterases) was increased in the starving cells. Many genes for transcriptional regulators of different families (GntR, AraC, LysR, TetR) were induced under starvation. Forty and 24 genes related to two-component regulatory systems were up- and down-regulated under starvation, respectively. Some of extracytoplasmic function (ECF) sigma-factors (ECA0813, ECA1540) or their cognate anti-sigma-factors (ECA0233, ECA2086) were induced in adapted cells (Table 4 and S4 Table).

### Down-regulated genes in the cross-protected *Pba* phenotype

GO analysis of the 1038 down-regulated genes revealed 35 subcategories: 15 biological process subcategories, 5 cellular component subcategories, 15 molecular function subcategories (Fig 4 and S3 Table). The largest subcategories in the biological process category were translation (GO:0006412), carbohydrate metabolic process (GO:0005975), DNA-templated transcription (GO:0006351), phosphoenolpyruvate-dependent sugar phosphotransferase system (GO:0009401), cell division (GO:0051301), cell cycle (GO:0007049), tricarboxylic acid cycle (GO:0006099), tRNA wobble uridine modification (GO:0002098) which were represented by 1.05–3.95% of all down-regulated genes. In the cellular component category, the largest subcategories were plasma membrane (GO:0005886) and ribosome (GO:0005840) with 10.69% and 2.99% of all down-regulated genes, respectively.

In the molecular function category, ATP binding (GO:0005524), DNA binding (GO:0003677), structural constituent of ribosome (GO:0003735), magnesium ion binding (GO:0000287), ATPase activity (GO:0016887), rRNA binding (GO:0019843), flavin adenine dinucleotide binding (GO:0050660), electron carrier activity (GO:0009055), sequence-specific DNA binding transcription factor activity (GO:0003700), protein transporter activity (GO:0008565) were the most abundant GO terms, making up 1.35–11.75% of each cluster (S3 Table).

Sixteen KEGG pathways were significantly down-regulated in the cross-protected phenotype (30–80% of KEGG pathway genes): Metabolic pathways (eca01100), Oxidative phosphorylation (eca00190), Microbial metabolism in diverse environments (eca01120), Ribosome (eca03010), Carbon metabolism (eca01200), Fructose and mannose metabolism (eca00051), Bacterial secretion system (eca03070), Inositol phosphate metabolism (eca00562), Glycolysis/Gluconeogenesis (eca00010), Amino sugar and nucleotide sugar metabolism (eca00520), Citrate cycle (TCA cycle) (eca00020), Biosynthesis of secondary metabolites (eca01110), Sulfur metabolism (eca00920), Methane metabolism (eca00680), Starch and sucrose metabolism (eca00500), Glyoxylate and dicarboxylate metabolism (eca00630) (Table 3).

Down-regulated genes with manually assigned descriptions were involved in such pathways as *One-component system, *Amino acid metabolism/biosynthesis, *Cell division, *Chromosome condensation, *Transcriptional regulators, *Coronofacic acid synthesis, *Virulence, *Toxin-antitoxin, *Horizontally acquired islands, *Nitrogen metabolism, *Chaperone protein, *Transporters, *Stress proteins, *Antioxidant defense, *Protein metabolism, *Carbon metabolism, *Cytochrome B- and C-type biogenesis, *Cell envelope biogenesis, *Membrane proteins, *Phage-like proteins, *Type IV secretion system, *Sigma-anti-sigma factors, *Other proteins, *Uncharacterized proteins and proteins of unknown function, *Other transcripts (S5 Table).

Thus, by functional classification of down-regulated genes, the following functional gene modules and pathways were found to be repressed in the cross-protected *Pba* phenotype: genes related to oxidative phosphorylation, metabolism of carbon and carbohydrates (including TCA cycle), sulfur, nucleotide, lipid, biosynthesis of secondary metabolites, processes of translation and protein modification. Herewith, RNA-Seq did not indicate on the decrease in general level of transcription; however, many genes for transcriptional regulators were among DEGs. Some genes related to amino acid metabolism were down-regulated, however pathways for methionine (S-adenosyl methionine) and arginine were up-regulated (Table 4 and S5 Table).

Most of the genes encoding virulence factors (plant cell wall degrading enzymes as well as other proteins transported to the periplasm *via* the Sec pathway and secreted proteases) were down-regulated in adapted cells. Virulence genes of *cfa*-like cluster (encoding synthases of coronafacic acid) located within HAI-2 were also down-regulated. Genes for motility and flagellar assembly proteins were down-regulated in cross-protective phenotype. However, many genes assigned to chemotaxis and flagellar motor were up-regulated (Table 4, S4 Table and S5 Table).

Many genes related to DNA repair (eca03430 –mismatch repair, eca03400 –DNA repair and recombination, eca03440 –homologous recombination) were repressed in adapted cells. The expression level of genes of SOS-regulon did not vary significantly in growing and adapted cells (Table 4 and S5 Table).

Many ABC transport uptake systems were down-regulated under starvation. However, several genes for transporters of metal cation (zinc, iron) and phosphorus and proteins related to amino acid transport were up-regulated under starvation (Table 4, S4 Table and S5 Table). Genes encoding well-known regulators of stress response (RelA, SpoT and RpoS) that are induced in *Pba* at early stages (4–8 h) of starvation and later their expression decreases to initial level (Petrova et al., 2014) were not among differentially expressed genes (S1 Table).

## Discussion

In the present study, we determined the period necessary for the formation of cross-protected phenotype in *Pba* under starvation conditions and portrayed physiological features of cells that acquired resistance to various stress factors compared to unstressed growing cells applying transcriptome analysis using RNA-Seq approach. Under starvation, *Pba*, as with many other bacterial species [33–35], becomes resistant to a number of stressors. In the present study, we additionally show that in *Pba*, maximal level of cross-protection is reached within 24 hour of starvation and does not increase further. Within this period, a transition from non-adaptive to adaptive physiological status is achieved and bacteria may persist under stress effect. Transcriptome analysis revealed 610 up-regulated and 1038 down-regulated genes in the cross-protected phenotype versus growing cells. These DEGs were grouped into functional modules and categories by KEGG database, GO enrichment analysis and manual classification using various databases. RNA-Seq results were verified by means of qRT-PCR and the data obtained by two methods showed good correlation.

The formation of cross-protected phenotype is known to require the action of global stress-induced regulators, particularly alarmone (p)ppGpp necessary for the induction of the stringent response and synthesized by RelA or SpoT and stress-induced sigma-factor RpoS [36]. The induction of expression of *relA*, *spoT* and *rpoS* genes under stress conditions in *Pba* and many other bacteria is known to occur during first few hours after stress exposure; thereafter their expression reaches the initial levels [7, 25, 37]. Here we show that maximal cross-protective effect does not coincide in time with the highest expression level of *relA* and *rpoS* and occurs when transcript level of these genes reaches the degree of unstressed cells [25 and this study]. Thus, the induction of *relA*, *spoT* and *rpoS* may be attributed to the alarm stage of stress response when cells are preparing to become resistant, and this stage precedes and triggers the resistance phase when the cross-protection is achieved. It is reasonable to note that the effects of ppGpp and RpoS (increase membrane stability, synthesis of glucose/trehalose and glycogen, activation of lipolytic activity and oxidation of fatty acids, etc.) resemble the reactions that are induced by animal stress hormones during the alarm stage of the stress response [38–45].

Our work allowed determination of several physiological features reflected in the transcriptome profile inherent to *Pba* cross-protective phenotype. General metabolic activity was evident to be lower in adapted cells than in growing ones since genes for oxidative phosphorylation, carbon and carbohydrate metabolism (including TCA cycle) and processes related to translation and protein modification were significantly repressed. Some genes related to amino acid metabolism were also down-regulated. However, several amino acid pathways were induced under starvation. Arginine biosyntesis pathway that includes genes for arginine and citruline metabolism was up-regulated in cross-protected phenotype. Additionally, the activation of formation of methionine and its derivatives from homocystein as well as methionine salvage cycle that is a universal pathway recycling sulfur-containing metabolites to methionine, occurred under starvation. It was previously shown, that arginine increased the level of c-di-GMP in *Salmonella* by modulating the expression of diguanilate cyclase gene that is necessary for the formation of biofilms under starvation condition [46–47]. The increase in citrulline content is known to result in the formation cross-protection in starving bacteria [48]. The role of methionine in bacterial adaptation is unknown. However, it is reasonable to hypothesize that the induction of methionone metabolism, including the methionine cycle, where this amino acid is converted to S-adenosyl methionine (SAM)–a principal biological methyl donor for numerous prokaryotic methyltranaferases, is related to the activation of the methylation processes that are evident from the RNA-Seq analysis as up-regulation of various methyltransferase genes in adapted *Pba* cells.

The maintenance of the cross-protective phenotype requires the presence of specific defensive proteins that support cell homeostasis under adverse conditions. The RNA-Seq analysis revealed the induction of several protective genes, including those for antioxidant defense. This is in accordance with the up-regulation of oxidative stress regulon, including the gene encoding catalase, in cross-protected *E*. *coli* phenotype [49]. Additionally, many genes related to biofilm formation, including those that encode regulators YbaJ and BssS, and diguanylate phosphodiesterases and diguanylate cyclases engaged in the synthesis of cyclo-di-GMP that was shown to induce biofilm formation [30], were activated under starvation, as well as genes related to the synthesis of capsule polysaccharides.

Although virulence genes such as those encoding the *cfa*-cluster, plant cell wall degrading enzymes and type III secretion system, were repressed in adapted cells, their transcripts were observed under starvation pointing to the “readiness” of bacteria to switch on virulence program fast when it is required. This is in accordance with the preservation of virulence of starving *Pba* cells shown in our previous study [25].

Cross-protected phenotype was characterized by the up-regulation of chemotaxis-related genes and genes for the flagellar stator motor. However, genes for motility and flagellar assembly proteins were repressed. This likely means that cross-protected cells do not spend exogenous resources for the synthesis of flagella *de novo*, however, they maintain activity of the flagellar motor and increase the sensitivity to chemoattractants to be able to “feel” the environment. This fact is in accordance with the ultrastructural changes of starving *Pba* cell: under starvation the volume of the cytoplasm decreases significantly, however, cells seek to maintain sufficient cell volumes and considerable outer surfaces that presumably serve as peculiar cell antennas catching various external signals [25]. This probably may enable bacteria to “escape” the stress-factor. The activation of chemotaxis under starvation was also noted previously [50].

Most of the genes related to DNA repair were either non-differentially expressed or down-regulated in adapted cells pointing to the possibility of the increase in mutation process in the starving bacterial population. Genes that are classified into a group of prokaryotic defense systems protecting cell from foreign DNA were differentially expressed in growing and starving cells. These defense systems are divided into two broad groups that differ in their modes of action [51]. The defense systems that function on the self-non-self discrimination principle constitute the first group that includes restriction-modification (RM) system, DND system (which labels DNA by phosphothiolation and destroys unmodified) and CRISPR (Clustered Regularly Interspaced Short Palindromic Repeats)-Cas (CRISPR-associated genes) system–a prokaryotic adaptive immunity system able to memorize encounters with foreign DNA and attack it specifically. Several RM- and DND-related genes were up-regulated in the starving cells. However, the expression of CRISPR-Cas locus was down-regulated. Toxin-antitoxin (TA) systems acting as inducers of programmed cell death or dormancy represent the second group of prokaryotic defense systems. In starving cells, 18 genes related to TA-systems were up-regulated.

Additionally, significant portion of genes within HAI7 where the Type IV secretion system and the rest of the conjugative functions annotated as an “integrated plasmid” are located [27, 31], as well as genes of HAI2 and HAI13 encoding conjugation-related genes were up-regulated in adapted cells. Thus, the obtained results show that although there were several up-regulated genes which products may protect cells from the foreign DNA, the whole transcriptome portray shows that in adapted cells, the background for mutational process, including DNA exchange through conjugation and existence of some foreign DNA, is formed. This likely enables bacteria to acquire new traits that may increase their survival under stress conditions.

It is obvious that the acquirement of cross-protection and the ability to maintain homeostasis at “novel level” requires switching on/off various regulatory systems. The RNA-Seq analysis revealed the regulatory systems that may coordinate physiological processes in adapted cells. These systems include multiple one- and two-component regulatory systems, transcriptional factors, sigma and anti-sigma factors and genes which products are involved in the synthesis of cyclo-di-GMP–a regulatory signal that is involved in particular in stress reactions of microorganisms. Additionally, in our recent study various small RNAs that are induced under starvation and potentially have regulatory effect on the formation of cross-protective phenotype were unraveled [52].

### Summary and outlook

Cross-protected cell phenotype of *Pba* formed under stress (starvation) conditions at the resistance stage of stress response is characterized by multiple physiological features reflected in the trascriptome profile that distinguish it from unstressed cells. The described alterations in the expression of gene modules and pathways likely enable cross-protected cells to maintain their resistance under prolonged stress effect. Stress response is known to be expressed in two types of adaptive strategies: “fight”–to resist stress factor, or “flight” to escape the stress factor. The RNA-Seq analysis of growing and cross-protected *Pba* cells indicated on several physiological parameters that may contribute to maintain the ability of adapted *Pba* cells to both “fight” the stress factor (e.g. expression of defense genes, decrease of general metabolism, increased mutational background, biofilm formation) and to “flight” to better life (e.g. increase in chemotaxis, the presence of transcripts of virulence genes). Herewith, proteins (or other metabolites) that might have been synthesized during the alarm stage of the stress response and preserved in the cells may also play roles in determination of the physiology of cross-protected *Pba* phenotype, though the encoding genes were not described as up-regulated in the cross-protected *Pba* phenotype in our study. Additionally, transcriptome analysis enabled determination of potential regulators involved in the promotion and/or maintenance of cross-protective features under adverse conditions.

For future research work on the described phenomenon, it would be informative to elucidate general and species-specific aspects of stress-induced resistance by comparing transcriptomes of cross-protected cells of bacteria belonging to different ecological groups. Additionally, since bacteria are able to resist stressors applying alternative adaptive reactions, many of which lead to the formation of cross-protection [25, 26], it would be interesting to compare transcriptome profiles of cross-protected phenotypes formed in the course of realization of different adaptive strategies. Valuable information may be also obtained by analyzing stress-induced transcriptomes of mutant bacteria that are unable to form cross-protection phenotypes under stress conditions (e.g. *rpoS*-, *relA*-deficient mutants). In addition, the analysis of mutants deficient in regulatory genes that were shown to be induced in the cross-protected *Pba* phenotype in our study, would allow verification of the roles played by these regulators in cell resistance to multiple stressors.

## Materials and Methods

### Bacterial strains, media and culture conditions

A strain of *P*. *atrosepticum* SCRI1043 (Bell et al., 2004), was used in this study. The cultures with inoculation titer of 2–3 × 10^6^ CFU (colony forming units) per ml were grown in Luria-Bertani medium [53], with aeration (200 r.p.m.) at 28°C for 16 h (growth-promoting conditions). Aliquots of these cultures were used for total RNA extraction. The remaining cells were transferred (after double wash) to carbon and phosphorus deficient AB medium containing 1 g/L NH_4_Cl; 0.62 g/L MgSO_4_ × 7H_2_O; 0.15 g/L KCl; 0.013 g/L CaCl_2_ × 2H_2_O, pH 7.5 and incubated under starvation conditions with initial population density of 5.4 × 10^8^ ± 6.1 × 10^7^ CFU per ml in glass vials without aeration at 28°C.

### Cross-protection assay

To assess the dynamics of the cross-protective effect of starvation stress (primary stress), the tolerance of starving (or non-starving) *Pba* cells toward heat shock, hydrogen peroxide and NaCl (secondary stress) was examined. For secondary stress challenge, 1 ml aliquots of cell suspensions incubated under starvation for 0, 4, 8, 24, and 48 h were subjected to 50°C for 5 min or supplemented with 2.5 mM H_2_O_2_ or 20% NaCl for 1 hour. Before and right after exposure to one of the secondary stress factors (heat shock, or H_2_O_2_, or NaCl), suspensions were plated onto 1.5% LB agar as serial 10-fold dilutions. Plates were incubated at 28°C for two days and the CFUs were counted. The survival of starving cell was assessed by the comparison of cell titer prior and after secondary stress factor exposure.

### Total RNA preparation

Total RNA was isolated from bacterial cells using the RNeasy Protect Bacteria Mini Kit (Qiagen, USA), according to the manufacturer’s instructions. Contaminating DNA was removed from the samples by DNAse (Qiagen) treatment. RNA was quantified using a Qubit fluorometer (Invitrogen, USA).

### cDNA library construction and bacteria strand-specific RNA sequencing

Library construction and strand-specific sequencing were carried out at the Beijing Genomics Institute (BGI-Shenzhen, China; http://www.genomics.cn/en/index), following the manufacturer’s protocols. Briefly, the rRNA was depleted from 1 microgram of total RNA using the Ribo-Zero Magnetic Gold Kit (Epicenter). TruSeq RNA Sample Prep Kit v2 (Illumina) was used for library construction. RNA was fragmented into small pieces using Elute Prime Fragment Mix. First-strand cDNA was synthesized with First Strand Master Mix and Super Script II (Invitrogen) reverse transcription (25°C for 10 min; 42°C for 50 min; 70°C for 15 min). After product purification (Agencourt RNAClean XP Beads, AGENCOURT) the second-strand cDNA library was synthesized using Second Strand Master Mix and dATP, dGTP, dCTP, dUTP mix (1 hour at 16°C). Purified fragmented cDNA was end repaired (30 min at 30°C) and purified with AMPureXP Beads (AGENCOURT). Addition of the poly (A) tail was done with A-tailing Mix (30 min at 37°C) prior to ligating sequencing adapters (10 min at 30°C). The second-strand cDNA was degraded using the Uracil-N-Glycosylase (UNG) enzyme (10 min at 37°C) and the product purified by AMPureXP Beads (AGENCOURT). Several rounds of PCR amplification with PCR Primer Cocktail were performed to enrich the cDNA fragments and the PCR products were purified with AMPureXP Beads (AGENCOURT). Sequencing was performed using the Illumina HiSeq^TM^ 2000 platform with pair-end 90 base reads. The sequencing data used in this study can be accessed from NCBI’s Gene Expression Omnibus, under the accession number GSE68547.

### RNA-seq data analysis

Strand-specific RNA-seq was used to compare whole transcriptomes of *Pba* cells under nutrient-rich and starvation conditions. Paired-end read quality control, trimming, read mapping and indexing of resulting BAM files, were performed as reported in Kwenda et al [52]. Gene quantification was conducted using bedtools multicov [54], to make counts of reads mapped to *Pba* protein-coding regions. In order to determine differentially expressed genes, edgeR package was used [55]. A false discovery rate (FDR) of 5% was used as cut-off for significantly differentially expressed genes.

### Assignment of differentially expressed genes to different gene modules, pathways and categories

KEGG pathways and Gene ontology IDs were collect from KEGG http://www.genome.jp/kegg/pathway.html and GO http://geneontology.org/page/go-database databases. The modified Fisher Exact test was used to determine whether the proportions of those falling into each KEGG or GO category differ by group as described in David database [56] (p-value≤0.05). Additional information about some genes was manually found using Uniprot http://www.genome.jp/kegg/pathway.html, MiST http://mistdb.com/, Pfam http://pfam.xfam.org/, PROSITE http://prosite.expasy.org/, ICEberg http://db-mml.sjtu.edu.cn/ICEberg/feature_page.php?ice_id=467, Ecogene http://ecogene.org/, String http://string-db.org/, TADB http://202.120.12.135/TADB2/index.php, dndDB http://db-mml.sjtu.edu.cn/dndDB/, SMART http://smart.embl-heidelberg.de/, HAMAP http://hamap.expasy.org/, EMBL-EBI http://www.ebi.ac.uk/services, REBASE http://rebase.neb.com/rebase/rebase.html, InterPro https://www.ebi.ac.uk/interpro/ and (Bell et al., 2004). Gene ontology categories were visualized using REVIGO http://revigo.irb.hr/.

### RT-qPCR validation of RNA-seq data

The verification of RNA-seq data was carried out by RT-real-time PCR. Fifteen up- and 15 down-regulated under starvation genes belonging to different pathways were chosen for the verification: up-regulated–ECA0999, ECA3238, ECA4082, ECA4065, ECA0049, ECA1065, ECA1216, ECA3920, ECA4477, ECA1059, ECA1166, ECA1540, ECA2013, ECA2873, ECA2226; down-regulated–ECA3054, ECA3682, ECA4006, ECA4513, ECA1141, ECA1232, ECA4461, ECA1407, ECA2651, ECA4079, ECA0260, ECA0255, ECA0238, ECA3343, ECA3814.

Total RNA was extracted from *Pba* cells and digested with DNAze as described above and used for cDNA synthesis. The reaction mixture for reverse transcription contained 1 mM random hexamers, 300 mM dNTPs, 1x reverse transcriptase buffer, 100 U RevertAid reverse transcriptase (Thermo Scientific, USA). First, water and random hexamers were mixed with RNA and incubated for 5 min at 70°C and cooled immediately on ice. The other components were then added. Incubation was performed using a DNA Engine thermocycler (Bio-Rad, Hercules, CA, USA). Reverse transcription was performed as follows: 10 min at 25°C, followed by 1 h at 37°C and 10 min at 70°C. Two μl of a dilution (1/5) of cDNA were used as the template for qPCR.

qRT-PCR was performed using the 2.5x kit containing Taq-polymerase and corresponding buffer, dNTPs and Eva Green dye (Synthol, Russia). Primers (S6 Table) designed according to the genome sequence of *P*. *atrosepticum* SCRI1043 (NCBI Gen- Bank accession number NC_004547) using Vector-NTI Version 9 software (Invitrogen, Carlsbad, CA, USA) and synthesized by Evrogen (Moscow, Russia) were added to 0.4 μM. PCR was performed under the following conditions: 94°C for 2 min, followed by 45 cycles at 94°C for 10 s, 60°C for 15 s and 72°C for 30 s. After PCR, the melt curve analysis (60–95°C) was performed. The reactions were run and changes in fluorescence emission were detected using a CFX96 quantitative PCR system (Bio-Rad, USA). The amount of fluorescence was plotted as a function of the PCR cycle using CFX Manager Software (Bio-Rad, USA). The amplification efficiency for all primers was determined using a dilution series of a pool of cDNAs. Additional controls included the omission of reverse transcriptase to measure the extent of residual genomic DNA contamination and omission of template.

The expression level of target genes was calculated relative to reference genes. The geNorm software was used to choose genes that displayed stability of expression under the experimental conditions. Among the candidate reference genes (*groES*, *recA*, *rpoD*, *ffh* and *gyrB*), the latter three had the most stable expression levels (M value– 0.862, 0.757 and 0.652, respectively; Pairwise Variations– 0.256). These genes were also among stably expressed according to RNA-Sed data. Therefore, *rpoD*, *ffh* and *gyrB* genes were used as reference ones. Relative expression levels were determined as the ratio between the quantity of cDNA corresponding to the target and reference gene transcripts. The data are presented as the average ± SD of five biological replicates. The correlation between the data obtained by RNA-Seq and qPCR was assessed by Spearman test.

## Supporting Information

S1 Table Relative expression levels of genes in adapted *P*. *atrosepticum* cells vs. growing ones. (XLS)

S2 Table GO analysis of *P*. *atrosepticum* genes up-regulated in adapted cells compared to growing ones (p-value≤0.05). (XLS)

S3 Table GO analysis of *P*. *atrosepticum* genes down-regulated in adapted cells compared to growing ones (p-value≤0.05). (XLS)

S4 Table Modules and pathways of *P*. *atrosepticum* genes up-regulated in adapted cells compared to growing ones. (XLS)

S5 Table Modules and pathways of *P*. *atrosepticum* genes down-regulated in adapted cells compared to growing ones. (XLS)

S6 Table Primers used in this study. (XLS)

## References

[1] Steinberg C, Sturzenbaum S, Menzel R. Genes and environment–striking the fine balance between sophisticated biomonitoring and true functional environmental genomics. Sci Total Environ. 2008; 400: 142–161. 10.1016/j.scitotenv.2008.07.023 18817948

[2] Stojanovich L. Stress and autoimmunity. Autoimmun Rev. 2010; 9: A271–A276. 10.1016/j.autrev.2009.11.014 19931651

[3] Renzone G, D’Ambrosio C, Arena S, Rullo R, Ledda L, Ferrara L, et al Differential proteomic analysis in the study of prokaryotes stress resistance. Ann Ist Super Sanita. 2005; 41: 459–468. 16569914

[4] Klauck E, Hengge R. Ϭ^S^-controlling networks in *Escherichia coli* In Filloux A, editor. Bacterial regulatory networks. Caister Academic Press Norfolk, UK; 2012 pp. 1–25

[5] Potrykus K., Cashel M. Preferential cellular accumulation of ppGpp or pppGpp in *Escherichia coli* In: Bruijn F, editor. Stress and environmental regulation of gene expression and adaptation in bacteria. John Wiley & Sons New York, US; 2016 pp. 479–485

[6] Kjelleberg S, Humhrey B, Marshall A. Initial phases of starvation and activity of bacteria at surfaces. Appl Environ Microb. 1983; 46: 978–984. 10.1128/aem.46.5.978-984.1983 PMC239507 16346433

[7] Battesti A, Majdalani N, Gottesman S. The RpoS-mediated general stress response in *Escherichia coli*. Annu Rev Microbiol. 2011; 65: 189–213. 10.1146/annurev-micro-090110-102946 21639793 PMC7356644

[8] Brockmann-Gretza O, Kalinowski J. Global gene expression during stringent response in *Corynebacterium glutamicum* in presence and absence of the *rel* gene encoding (p)ppGpp synthase. BMC Genomics. 2006; 7: 230 10.1186/1471-2164-7-230 16961923 PMC1578569

[9] Durfee T, Hansen A-M, Zhi H, Blattner F, Jin D. Transcription profiling of the stringent response in *Escherichia coli*. J Bacteriol. 2008; 190: 1084–1096. 10.1128/JB.01092-07 18039766 PMC2223561

[10] Brown D, Barton G, Pan Z, Buck M, Wigneshweraraj S. Combinatorial stress responses: direct coupling of two major stress responses in *Escherichia coli*. Microbial Cell. 2014; 9: 315–317. 10.15698/mic2014.09.168 PMC5349134 28357257

[11] Gill C. Microbial control with cold temperatures In: Juneja V, Sofos J, editors. Control of foodborne microorganisms. Taylor & Francis Group, Boca Raton; 2001 pp. 55–74

[12] Foster J, Spector M. How *Salmonella* survive against the odds. Annu Rev Microbiol. 1995; 49: 145–74. 10.1146/annurev.mi.49.100195.001045 8561457

[13] Chung H, Bang W, Drake M. Stress response of *Escherichia coli*. Compr Rev Food Sci Food Saf. 2006; 5: 52–64.

[14] Cozens R, Tuomanen E, Tosch W, Zakn O, Suter J, Tomasz A. Evaluation of the bactericidal activity of P-lactam antibiotics on slowly growing bacteria cultured in the chemostat. Antimicrob Agents Chemother. 1986; 29: 797–802. PMCID: PMC284156 3089141 10.1128/aac.29.5.797 PMC284156

[15] Jenkins D, Chaisson S, Matin A. Starvation-induced cross protection against osmotic challenge in *Escherichia coli*. J Bacteriol. 1990; 172: 2779–2781. 2185233 10.1128/jb.172.5.2779-2781.1990 PMC208926

[16] Jenkins D, Schultz J, Matin A. Starvation-induced cross protection against heat or H_2_O_2_ challenge in *Escherichia coli*. J Bacteriol. 1988; 170: 3910–3914. 3045081 10.1128/jb.170.9.3910-3914.1988 PMC211389

[17] Hong S, Mendonça AF, Daraba A, Shaw A. Radiation resistance and injury in starved *Escherichia coli* O157:H7 treated with electron-beam irradiation in 0.85% saline and in apple juice. Foodborne Pathog Dis. 2014; 11: 900–906. 10.1089/fpd.2014.1782 25393670

[18] Bocsanczy A, Achenbach U, Mangravita-Novo A, Chow M, Norman D. Proteomic comparison of *Ralstonia solanacearum* strains reveals temperature dependent virulence factors BMC Genomics. 2014; 15: 280 10.1186/1471-2164-15-280 24725348 PMC4023598

[19] Perombelon M. Potato diseases caused by soft rot erwinias: an overview of pathogenesis. Plant Pathol. 2002; 51: 1–12.

[20] Mole B, Baltrus D, Dangl J, Grant S. Global virulence regulation networks in phytopathogenic bacteria. Trend in microbiology. 2007; 14: 363–371. 10.1016/j.tim.2007.06.005 17627825

[21] Lavelle P. Soil as a Habitat. In: Wall D, editor Soil Ecology and Ecosystem Services. Oxford University Press; 2012 pp. 7–27

[22] Charkowski A, Blanco C, Condemine G, Expert D, Franza T, Hayes C. et al The role of secretion systems and small molecules in soft-rot *Enterobacteriaceae* pathogenicity. Annu Rev Phytopathol. 2012; 50: 425–449. 10.1146/annurev-phyto-081211-173013 22702350

[23] Gorshkov V, Petrova O, Mukhametshina N, Ageeva M, Mulyukin A, Gogolev Y. Formation of “nonculturable” dormant forms of the phytopathogenic enterobacterium *Erwinia carotovora*. Microbiology (Mosc). 2009; 78: 585–592.

[24] Gorshkov V, Petrova O, Gogoleva N, Gogolev Y. Cell-to-cell communication in the populations of enterobacterium *Erwinia carotovora* ssp. *atroseptica* SCRI1043 during adaptation to stress conditions. FEMS Immunol Med Microbiol. 2010; 59: 378–385. 10.1111/j.1574-695X.2010.00684.x 20528924

[25] Petrova O, Gorshkov V, Daminova A, Ageeva M, Moleleki L, Gogolev Y. Stress response in *Pectobacterium atrosepticum* SCRI1043 under starvation conditions: adaptive reactions at a low population density. Res Microbiol. 2014; 165: 119–127. 10.1016/j.resmic.2013.11.004 24300393

[26] Petrova O, Gorshkov V, Sergeeva I, Daminova A, Ageeva M, Gogolev Y. Alternative scenarios of starvation-induced adaptation in *Pectobacterium atrosepticum*. Res Microbiol. 2016; 167: 254–261. 10.1016/j.resmic.2016.01.009 26912323

[27] Bell KS, Sebaihia M, Pritchard L, Holden MTG, Hyman LJ, Holeva MC, et al Genome sequence of the enterobacterial phytopathogen *Erwinia carotovora* subsp. *atroseptica* and characterization of virulence factors. PNAS. 2004; 101: 11105–11110. 10.1073/pnas.0402424101 15263089 PMC503747

[28] Domka J, Lee J, Wood T. YliH (BssR) and YceP (BssS) regulate *Escherichia coli* K-12 biofilm formation by influencing cell signaling. Appl Environ Microbiol. 2006; 72: 2449–2459. 10.1128/AEM.72.4.2449-2459.2006 16597943 PMC1448992

[29] Garcia-Contreras R, Zhang X-S, Kim Y, Wood T. Protein translation and cell death: the role of rare t-RNAs in biofilm formation and in activating dormant phage killer genes. PLoS ONE. 2008; 3: e2394 10.1371/journal.pone.0002394 18545668 PMC2408971

[30] Pérez-Mendoza D, Coulthurst S, Sanjuán J, Salmond G. N-Acetylglucosamine-dependent biofilm formation in *Pectobacterium atrosepticum* is cryptic and activated by elevated c-di-GMP levels. Microbiology. 2011; 157: 3340–3348. 10.1099/mic.0.050450-0 21948048

[31] Evans T, Pérez-Mendoza D, Monson R, Stickland H, Salmond G. Secretion systems of the enterobacterial phytopathogen, *Erwinia* In: Wooldridge K, editor. Bacterial secreted proteins: secretory mechanisms and role in pathogenesis. Caister Academic Press Norfolk, UK; 2009 pp. 479–502

[32] Pritchard L, Liu H, Boot C, Douglas E, Francois P, Schrenzel J, et al Microarray comparative genomic hybridization analysis incorporating genomic organization, and application to enterobacterial plant pathogens. PLOS Comput Biol. 2009; 5, e10000473. 10.1371/journal.pcbi.1000473 PMC2718846 19696881

[33] García S, Limón J, Heredia N. Cross protection by heat and cold shock to lethal temperatures in *Clostridium perfringens*. Braz J Microbiol. 2001; 32: 110–112

[34] Siddiqui I, Shaukat S. Response to carbon-starvation in *Pseudomonas aeruginosa* strain IE-6S+: analysis of general cross protection, production of some nematocidal compounds in vitro, and the biological control of *Meloidogyne javanica* in tomato. World J Microbiol Biotechnol. 2003; 19: 917–924.

[35] den Besten H, Mols M, Moezelaar R, Zwietering M, Abee T. Phenotypic and transcriptomic analyses of mildly and severely salt-stressed *Bacillus cereus* ATCC 14579 cells. Appl Environ Microbiol. 2009; 75: 4111–4119. 10.1128/AEM.02891-08 19395575 PMC2698350

[36] Behmardi P, Grewal E, Kim Y, Yang H. RpoS-dependant mechanism is required for cross-protection conferred to hyperosmolarity by heat shock. J Exp Microbiol Immunol. 2009; 13: 18–21

[37] Morton D, Oliver J. Induction of carbon starvation-induced proteins in *Vibrio vulnificus*. Appl Environ Microbiol. 1994; 60: 3653–3659. 16349411 10.1128/aem.60.10.3653-3659.1994 PMC201869

[38] de Gomez Dumm I, de Alaniz M, Brenner R. Effect of glucocorticoids on the oxidative desaturation of fatty acids by rat liver microsomes. J Lip Res. 1979; 20: 834–839. 490055

[39] Hengge-Aronis R, Klein W, Lange R, Rimmele M, Boos W. Trehalose synthesis genes are controlled by the putative sigma factor encoded by *rpoS* and are involved in stationary-phase thermotolerante in *Escherichia coli*. J Bacteriol. 1991; 173: 7918–7924. 1744047 10.1128/jb.173.24.7918-7924.1991 PMC212585

[40] Pitzalis C, Pipitone N, Perretti M. Glucocorticoids and leukocyte adhesion In: Goulding N, Flower R, editors. Glucocorticoids. Basel. Boston. Berlin: Birkhauser Verlag; 2001 pp. 105–118

[41] Broich M, Rydzewski K, McNealy T, Marre R, Flieger A. The global regulatory proteins LetA and RpoS control phospholipase A, lysophospholipase A, acyltransferase, and other hydrolytic activities of *Legionella pneumonia* JR32. J Bacteriol. 2006; 188: 1218–1226. 10.1128/JB.188.4.1218-1226.2006 16452402 PMC1367211

[42] Xu C, He J, Jiang H, Zu L, Zhai W, Pu S, et al Direct effect of glucocorticoids on lipolysis in apidocytes. Mol Endocrinol. 2009; 23: 1161–1170. 10.1210/me.2008-0464 19443609 PMC5419195

[43] Wilson W, Roach P, Montero M, Baroja-Fernandez E, Murios F, Eydallin G, et al Regulation of glycogen metabolism in yeast and bacteria. FEMS Microbiol Rev. 2010; 34: 952–985. 10.1111/j.1574-6976.2010.00220.x 20412306 PMC2927715

[44] Charoenwong D, Andrews S, Mackey B. Role of *rpoS* in the development of cell envelope resilience and pressure resistance in stationary-phase *Escherichia coli*. Appl Environ Microbiol. 2011; 77: 5220–5229. 10.1128/AEM.00648-11 21705547 PMC3147466

[45] Kuo T, McQueen A, Chen T, Wang J. Regulation of glucose homeostasis by glucocorticoids. Adv Exp Med Biol. 2015; 872: 99–126. 10.1007/978-1-4939-2895-8_5 26215992 PMC6185996

[46] Mills E, Petersen E, Kulasekara B, Miller S. A direct screen for c-di-GMP modulators reveals a *Salmonella typhimurium* periplasmic L-arginine-sensing pathway. Sci Signal. 2015; 8: ra57 10.1126/scisignal.aaa1796 26060330

[47] Hengge R, Grundling A, Jenal U, Ryan R, Yildis F. Bacterial signal transduction by c-di-GMP and other nucleotide second messengers. J Bacteriol. 2016; 198: 15–26. 10.1128/JB.00331-15 26055111 PMC4686208

[48] Cusumano Z, Caparon M. Citrulline protects *Streptococcus pyogenes* from acid stress using the arginine deiminase pathway and the F_1_F_0_-ATPase. J Bacteriol. 2015; 197: 1288–1296. 10.1128/JB.02517-14 25645553 PMC4352666

[49] Gunasekera T., Csonka L., Paliy O. Genome-wide transcriptional responses of *Escherichia coli* K-12 to continuous osmotic and heat stresses. J Bacteriol. 2008; 190: 3712–3720. 10.1128/JB.01990-07 18359805 PMC2395010

[50] Wei X, Bauer W. Starvation-induced changes in motility, chemotaxis, and flagellation of *Rhizobium meliloti*. Appl Environ Microbiol. 1998; 64; 1708–1714. 9572940 10.1128/aem.64.5.1708-1714.1998 PMC106219

[51] Makarova K, Wolf Y, Koonin E. Comparative genomics of defense systems in archaea and bacteria. Nucleic Acids Res. 2013; 41: 4360–4377. 10.1093/nar/gkt157 23470997 PMC3632139

[52] Kwenda S, Gorshkov V, Ramesh A, Naidoo S, Rubagotti E, Birch P, et al Discovery and profiling of small RNAs responsive to stress conditions in the plant pathogen *Pectobacterium atrosepticum*. BMC Genomics. 2016; 17:47 Huang da W, Sherman B, Lempicki R. Systematic and integrative analysis of large gene lists using DAVID bioinformatics resources. Nat Protoc. 2009; 4: 44–57. 10.1186/s12864-016-2376-0 26753530 PMC4710047

[53] Sambrook J, Fritsch E, Maniatis T. Molecular Cloning: A Laboratory Manual. 2nd ed., Cold Spring Harbor, NY: Cold Spring Harbor Laboratory Press; 1989

[54] Quinlan A, Hall I. BEDTools: a flexible suite of utilities for comparing genomic features. Bioinformatics. 2010; 26: 841–842. 10.1093/bioinformatics/btq033 20110278 PMC2832824

[55] Robinson M, McCarthy D, Smyth G. edgeR: a Bioconductor package for differential expression analysis of digital gene expression data. Bioinformatics. 2010; 26: 139–140. 10.1093/bioinformatics/btp616 19910308 PMC2796818

[56] Huang da W, Sherman B, Lempicki R. Systematic and integrative analysis of large gene lists using DAVID bioinformatics resources. Nat Protoc. 2009; 4: 44–57. 10.1038/nprot.2008.211 19131956

